# Of Blickets, Butterflies, and Baby Dinosaurs: Children’s Diagnostic Reasoning Across Domains

**DOI:** 10.3389/fpsyg.2020.02210

**Published:** 2020-08-25

**Authors:** Deena Skolnick Weisberg, Elysia Choi, David M. Sobel

**Affiliations:** ^1^Department of Psychological and Brain Sciences, Villanova University, Villanova, PA, United States; ^2^Department of Applied Psychology, New York University, New York, NY, United States; ^3^Department of Cognitive, Linguistic, and Psychological Sciences, Brown University, Providence, RI, United States

**Keywords:** diagnostic inference, scientific reasoning, early elementary school, causal reasoning, contextualization

## Abstract

The three studies presented here examine children’s ability to make diagnostic inferences about an interactive causal structure across different domains. Previous work has shown that children’s abilities to make diagnostic inferences about a physical system develops between the ages of 5 and 8. Experiments 1 (*N* = 242) and 2 (*N* = 112) replicate this work with 4- to 10-year-olds and demonstrate that this developmental trajectory is preserved when children reason about a closely matched biological system. Unlike Experiments 1 and 2, Experiment 3 (*N* = 110) demonstrates that children struggle to make similar inferences when presented with a parallel task about category membership in biology. These results suggest that children might have the basic capacity for diagnostic inference at relatively early ages, but that the content of the inference task might interfere with their ability to demonstrate such capacities.

## Introduction

Diagnostic reasoning — inferring causes from systematic observations of patterns of data — is a hallmark of scientific thinking. It involves reasoning backwards, often from observed effects to potential causes. If our car doesn’t start, we wonder if there is fuel in the tank. If we smell gas in our apartment, we check the stove to find out whether there is a leak. If a soufflé doesn’t rise, we wonder whether we added enough cream of tartar. As adults, we easily engage in this kind of diagnostic reasoning (e.g., Thomas et al., 2008; Fernbach et al., 2010, 2011), which can be construed as a form of causal reasoning (Gopnik et al., 2001).

Research on children’s causal reasoning shows that preschoolers, and in some cases infants, can diagnose causal structures, draw inferences about the nature of causal systems from observed data, and even explore in systematic ways that both reflect appropriate causal inferences and generate new pieces of knowledge (Gopnik et al., 2004; Koerber et al., 2005; Sobel and Kirkham, 2006; Schulz and Bonawitz, 2007; Cook et al., 2011). Although this work seems to suggest that diagnostic reasoning is present early, in all of these studies, children or babies observe the efficacy of all or all but one potential cause in a system. Children’s thinking shows a more pronounced developmental trajectory when asked to reason about systems with multiple uncertain causes (Beck et al., 2006; Fernbach et al., 2012; Erb and Sobel, 2014; Sobel et al., 2017).

As an example, Sobel et al. (2017) showed 5- to 8-year-olds a pattern of data generated by an interactive causal model where the efficacy of some causes was left unspecified. Specifically, children in this study were asked to reason about a blicket detector machine (Gopnik and Sobel, 2000): a box that lights up and plays music when objects like blocks are placed on it, giving the illusion that the blocks make the box light up. Children were shown four possible causes (different blocks) that could cause one of three different kinds of activation on the machine (off, red, or green with music). In this system, two of the blocks are jointly necessary for the target effect (green with music), but these two blocks have a different effect (red) when they are not used together. This kind of reasoning problem parallels the inferences one would have to make in scientific reasoning; indeed, the causal structure of this blicket detector system was based on a model presented to older children in a test of scientific thinking called *Earthquake Forecaster* (Kuhn et al., 2009). Sobel et al. (2017) found clear developmental differences between 5 and 8 years in children’s reasoning about this system. The 5- and 6-year-olds in this study responded no differently from chance, while the 7- and 8-year-olds were successful at diagnosing the causal structure.

Engaging in diagnostic reasoning in tasks like this one requires a set of cognitive and metacognitive capacities, which have been well-documented in both the literature on cognitive development and on children’s developing scientific reasoning (Klahr, 2002) (see e.g., Zimmerman, 2000; Kuhn, 2007). But in addition to these general cognitive abilities, in order to diagnose potential causes, one may also need domain-specific knowledge in order to understand which events are potential causes of observed data. For example, to diagnose why our soufflé didn’t rise, we must know that not adding enough cream of tartar will cause a flat soufflé. We must contrast that possibility with the possibility that we curdled the eggs, overwhipped them, or failed to bring them to room temperature prior to whipping. To do this, we must know that these are also potential causes of flat soufflés (among numerous others). That is, we use our prior knowledge to form a hypothesis space in which we reason diagnostically (Sobel et al., 2004; Goodman et al., 2011; Griffiths et al., 2011; Ullman et al., 2012).

Given this, results from studies on infants and young children suggest that they have the cognitive capacity to engage in diagnostic reasoning only under optimal conditions, where they not only are presented with data sets that lack uncertainty but also where they are not required to bring more than the most general prior knowledge to bear on solving the problem. Indeed, the blicket detector paradigm, which is used in much of the prior work on young children’s causal reasoning abilities, asks children to reason about physical causal relations that they can see and articulate (Sobel and Munro, 2009a). This paradigm purposely aims to minimize and control the amount of prior knowledge that is necessary for children to successfully make diagnostic inferences (see, e.g., Schulz and Sommerville, 2006; Schulz and Bonawitz, 2007; Schulz et al., 2007; Sobel and Sommerville, 2009b; Bonawitz et al., 2011, for other examples).

It is thus an open question whether children are still successful at diagnostic reasoning when the context of the reasoning task is changed in a way that might affect how they construct the potential hypothesis space – specifically, by adding real-world information that might interact with the general reasoning abilities that children bring to bear in thinking about the task. Across three experiments, we investigated whether a task’s surface features – specifically whether those surface features seem to require the child to enlist their domain-specific knowledge – would affect children’s diagnostic inferences. Importantly, the structure of the reasoning problem presented in all of the current experiments was identical; no domain-specific biological knowledge was required to solve any of our tasks. However, because some versions of the task couched it in terms of biological systems, they may have encouraged children to bring such knowledge to bear on the problem. Our general goal was to investigate whether this would affect children’s reasoning abilities, either positively or negatively.

To do so, we first replicated the Sobel et al. (2017) procedure using a blicket detector with a wider age range and larger sample size. We also presented children with an analogous diagnostic reasoning problem with different surface features (butterflies and flowers). We compared these two tasks in both a between subject (Experiment 1) and a within-subject (Experiment 2) design. Our goal in these first two experiments was to see whether children would make the same kind of diagnostic inference in a domain other than physical causality and, if so, how those inferences compared. Results from these two experiments can thus illustrate whether minimal changes to the surface features of a diagnostic inference task might affect children’s reasoning abilities.

Experiment 3 moved beyond these tasks in several ways. We presented children with a diagnostic inference task that was about category membership, instead of about causal relations, but that had the same underlying structure as the systems in Experiments 1 and 2. Specifically, Experiment 3 asked children to make diagnostic inferences about category membership of a set of dinosaurs. Although the interactive structure was identical to the reasoning problems in Experiments 1 and 2, numerous aspects of the procedure differed. The task introduced potentially novel vocabulary (e.g., new dinosaur names) and embedded the problem in a more traditional scientific framework. Moreover, children had to track different features as potentially relevant, instead of different levels of a single feature (color in Experiments 1 and 2). All of these changes added to the information processing capacities necessary to make a diagnostic inference.

As such, Experiment 3 was deliberately designed to differ from Experiments 1 and 2 in a variety of ways. Our main goal in this experiment was not to investigate the impact of individual features or combinations of features on children’s performance in a diagnostic reasoning task. Rather, we aimed to translate our causal system into a form that more closely resembled scientific thinking problems that have been previously used to assess children’s reasoning abilities (e.g., Kuhn and Dean, 2005; see also Kuhn et al., 2009). Because children might encounter problems like this one in a classroom or a genuine science lab, this dinosaur version of the task serves as an important bridge between work with the blicket detector systems typically used in cognitive developmental psychology and work with the more realistic systems typically used in education science.

Experiment 3 can thus help us to determine whether children’s diagnostic reasoning capacities are robust and domain-general, in which case they should show a similar developmental trajectory to Experiments 1 and 2, or whether more realistic presentations of a diagnostic reasoning problem can affect their performance. That is, although the current design does not allow us to determine which particular demand characteristics may influence children’s reasoning, it does allow us to examine whether children possess domain-general diagnostic reasoning abilities or whether those abilities are limited by information processing demands or domain-specific knowledge. No prior work, to our knowledge, has presented a causal system previously studied with blicket detectors (as in developmental psychology) within a realistic scientific framework (as in education science), allowing this experiment to begin to build a bridge between these two literatures.

## Experiment 1

In Experiment 1, we replicated the procedure used by Sobel et al. (2017) and extended it to a second diagnostic reasoning problem with the same causal structure. This second problem – about whether butterflies were attracted to different colors of flowers – was designed to be as similar to the blicket detector version of the procedure as possible, but instead of asking about the physical relation between objects and a machine, it presented a task about biological mechanisms. Of interest is (a) whether we replicate the finding demonstrated by Sobel et al. (2017) that children begin to succeed at diagnostic inference tasks of this nature around age 7, and (b) whether children show similar development for a measure with different surface features, but the same underlying causal structure.

### Method

#### Participants

The final sample consisted of 242 children between the ages of 4 and 10 (118 boys, 124 girls, *M*_age_ = 83.09, SD = 21.54). This sample size was chosen to parallel the sample size of Sobel et al.(2017, Study 3). Ten additional children were tested, but not included in the final sample. Nine were excluded because of experimental error and one was not fluent in English. Children were tested at a local science museum and several preschools in the Philadelphia area. The racial breakdown of the sample was as follows: 153 families were White/Caucasian, 25 were Black/African American, 22 were of Asian descent, 1 was of Native American descent, 13 were of mixed descent and 28 did not report this information. The ethnic breakdown of the sample was as follows: 18 families reported as Hispanic, 133 reported as not Hispanic, and 91 families did not report this information.

#### Materials

**FIGURE 1:**
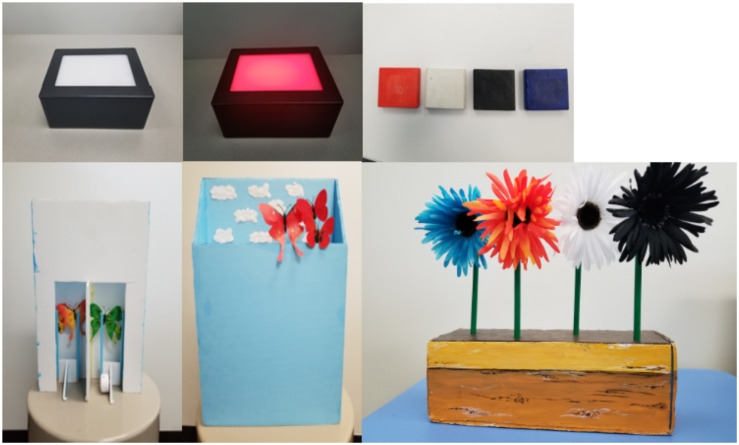
Stimuli for the blicket **(top)** and butterfly **(bottom)** conditions. The bottom left picture shows the back side of the butterfly apparatus. The experimenter would lift the correct color of butterflies upwards and the butterflies would appear to the participant as in the bottom middle picture. Because the green butterflies also were accompanied by music, a speaker that plays music when pressed is attached to the left side of handle.

We used a blicket detector, which is a remote-controlled, battery-powered rectangular box (19.5 cm × 15 cm × 7.8 cm). The box was black with a white pressure-sensitive plate on top (see Figure 1, top left panel). Pressing this plate would trigger a set of LED lights under it, which would make the machine turn on different colored lights. The machine could also play music from an internal speaker when activated. A second experimenter, who sat behind the first experimenter running the study, controlled the blicket using a hidden remote. This remote was used to first activate the machine (so that placing objects on the pressure-sensitive plate would make it turn on) and then was used to change the color of the activation (to red or to green, depending on the trial; see below).

This condition also used four small square canvases stretched over thick wooden frames (which we called “blocks”) painted white, blue, orange, and black (5 cm × 5 cm; see Figure 1, top right panel). There was also a clear cylindrical container in which the blocks could fit (height 8 cm, radius of base 4.25 cm). We also created a set of 7 laminated photographs of different combinations of the colored blocks (9.5 cm × 7.25 cm). These were used as visual reminders of each step of the procedure (4 photographs) and as possible responses to our test question (3 photographs; see Procedure section).

We also used four silk flowers in the colors white, black, orange, and blue. The flowers were all of the same type and size (12 cm radius) and were purchased from a local craft store. We glued these flowers onto wooden dowels, which were painted green in order to look like flower stems (20 cm). We constructed a flower pot in which to “plant” the flowers, using a rectangular block of foam (31 cm × 11 cm × 11 cm), which we painted yellow and brown (see Figure 1, bottom right panel). We punched holes in the top of the foam in order to “plant” the flowers.

We also used 6 plastic butterflies, 3 green and 3 red. The two sets of butterflies were identical except for their color. Two small butterflies in each color were 4.5 cm × 5.5 cm; one large butterfly in each color was 10 cm × 10 cm. We glued each set of butterflies onto wooden sticks, which were painted sky blue (20 cm). To display these stimuli, we constructed a box out of foam board. This box was rectangular with no top and with one side shorter than the other (see Figure 1, bottom left panel; front dimensions 30 cm × 33 cm, back dimensions 30 cm × 45 cm). The whole box was painted sky blue and white clouds were glued onto the inside to make it look like the butterflies were flying in the sky. We also used a commercially available sound device which could record and re-play a sound. This was placed at the back of the butterfly box where an experimenter could activate it out of view of the participant. Finally, as for the blicket version of the task, we made 7 laminated photographs of different combinations of the flowers (9.5 cm × 7.25 cm).

Both versions of the task also used a folded piece of cardboard as an occluder (approximately 60 cm × 100 cm) and 1 red and 2 green dots (1 cm in diameter).

#### Procedure

**FIGURE 2:**
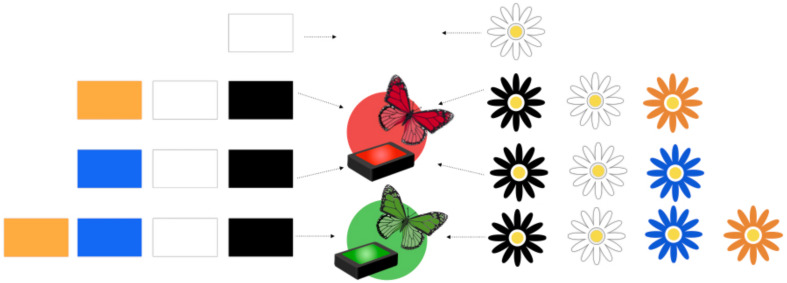
Schematic of Order 1 for the blicket and butterfly tasks. Each row shows the combination of blocks/flowers used in one step of the demonstration phase of the task.

Figure 2 shows a schematic of the data that were presented to children in this study. Children saw four demonstrations of how the blicket detector system/flower system worked. These four demonstrations were shown in one of two orders. Order 1 (shown in Figure 2) first demonstrated the effect of the white block/flower; then the effect of the combination of the white, black, and orange blocks/flowers; then the effect of the white, black, and blue blocks/flowers; then the effect of all four blocks/flowers. Order 2 presented these combinations in the opposite order (all four, then white and black and blue, then white and black and orange, then white alone). Children were randomly assigned to an order. Here, we use Order 1 to illustrate the procedure.

##### Blicket condition

First, the experimenter placed the white block in the clear container and put the container on top of the machine. She narrated her actions: “Let’s see what happens when I put just the white block on the machine”. The machine made no response, which the experimenter noted aloud: “Nothing happened.” She tried the white block again, again with no effect. She then brought out a photograph of the white block and put it on the table next to the machine, saying, “This is here to remind us what happened. When I put just the white block on the machine, nothing happened.”

Second, she put the white, black, and orange blocks in the container and put the container on the machine. The machine turned red, and the experimenter noted this: “Look, it’s turning red.” (Note that our use of the container ensured that all of the blocks contacted the machine’s surface at once, making it appear as though they were all jointly necessary for the effect.) The experimenter then tried the white, black, and orange blocks again, confirming that the machine turned red. She brought out the photograph of these three blocks and put a red dot next to it, saying, “Here’s a red dot to remind us that the machine turned red when we put the white, black, and orange blocks on the machine.” This same procedure was repeated for the combination of white, black, and blue blocks.

Finally, the experimenter put all four blocks in the container and set the container on top of the machine. This made the machine turn green and play music: “It’s going green and playing music!” She repeated this demonstration a second time. Then she put down the photograph of all four blocks on the table. She put a green dot beside this photograph and said, “Here’s a green dot to remind us that when we put the white, black, orange, and blue blocks on the machine, the machine turned green and made sound.” The four photographs and their accompanying dots remained on the table for the rest of the procedure as a visual reminder to children of the data they had observed.

After showing all four of these combinations, the experimenter put up the cardboard occluder between the child and the machine in order to prevent the child from seeing what objects were placed on the machine. The experimenter told the child that she was placing two blocks on the machine and that the machine was turning green. The child could confirm that the machine turned green because it was playing the music that they had heard when the machine turned green before. The experimenter repeated this activation, again playing the music and saying, “It’s turning green.”

The experimenter then took away the occluder and reminded the child that the machine had just turned green when she put two blocks on it. She set out three new photographs, each of which showed a pair of blocks: blue and black, orange and black, and orange and blue. Then, the experimenter asked the main test question: “Which two blocks on the machine made it go green?” The correct answer is orange and blue: The white block has no effect, the pairs of blue/black and of orange/black were previously shown to turn the machine red, and the machine turned green only when the orange and blue blocks were on the machine together.

After the participant chose one of the options, the experimenter asked why they chose it. Note that, in this condition, this question was included mid-way through data collection, so only 39 participants responded. Lastly, participants had the opportunity to try any combinations of blocks they wanted on the machine and to observe the effects.

##### Butterfly condition

This condition paralleled the procedure in the Blicket condition, using flowers instead of blocks and butterflies instead of lights. That is, instead of putting blocks in a container and placing the container on a machine to make it turn red or green, the experimenter “planted” different combinations of flowers in the foam block (“field”) and then placed the block next to the light blue box (“sky”). Instead of showing red or green lights, the experimenter made either the red or the green butterflies appear by lifting them on their stick to poke out above the front wall of the box apparatus (see bottom center panel of Figure 1). When the green butterflies appeared, she also pushed the button on the sound machine to make a song play. As in the Blicket condition, participants were provided with photographs of the four combinations of flowers, paired with green or red dots, as a visual reminder of what they had observed.

Participants were shown the same combinations of data and effects, in either Order 1 or Order 2 (see Figure 1). After observing the four demonstrations, the experimenter put up the occluder and said that she was planting two flowers in the field, which brought green butterflies. She activated the sound machine to make the sound that has previously been associated with the appearance of the green butterflies, so that participants could verify her claim, as in the Blicket condition. Then, they were asked the same test question as in the Blicket condition: “Which two flowers made the green butterflies come?” After responding to this question, all participants in this condition were asked to justify their response. Finally, as an exploratory investigation of children’s own actions on causal systems, they were also given the opportunity to plant whatever combination of flowers they wanted and observe the effects.

There were 116 children in the blicket condition and 126 children in the butterfly condition; there are fewer children in the blicket condition because errors with the detector led to more exclusions in this condition. Condition assignment was only partially random because we began by running most of the blicket condition first, which explains the discrepancy in the number of participants who were asked the justification question by condition. Participants in both conditions also engaged in an open-ended interview about the word “science” as part of a different investigation. Their responses to this question had no bearing on their performance in either the Blicket or Butterfly conditions and will not be discussed further.

#### Coding

In both conditions, we coded answers of blue/orange as correct and answers of black/orange or black/blue as incorrect. Children’s justifications were coded as to whether they were relevant to the task or irrelevant. Relevant justifications appealed to the data that the child had observed in the demonstration phase or to the fact that a particular combination of colors would be efficacious (e.g., “because gray and orange were in this when it made green”). Justifications that reflected the child’s beliefs but that did not provide any substantive information (e.g., “I just think it be the right ones,”) were coded as irrelevant. Justifications of “I don’t know” or cases in which children did not provide a justification were also coded as irrelevant. Justifications were coded by two naïve research assistants, blind to children’s age and gender. Agreement was 90% (Kappa = 0.79). Disagreements were resolved by discussion with the third author.

### Results

Table 1 shows performance on both the blicket and butterfly tasks across the age groups. Preliminary analysis showed that order of trials children received did not affect performance, χ^2^(1, *N* = 242) = 0.03, *p* = 0.85, so we did not analyze this factor further. There was also not a significant difference between girls’ and boys’ responses, χ^2^(1, *N* = 242) = 2.67, *p* = 0.11, so we did not consider this factor further. To analyze responses to the main test question, we constructed a general linear model with a binomial logistic distribution, analyzing the role of age (in months) and condition (blicket, butterfly) using a main effect only model (which proved more explanatory than a factorial model as evidenced by a lower BIC value^1^). The overall model was significant, χ^2^(2) = 11.33, *p* = 0.003. A main effect of age was found, Wald χ^2^(1) = 10.56, *p* = 0.001. No effect of condition was found, Wald χ^2^(1) = 0.23, *p* = 0.63.

**TABLE 1:** Proportion of correct responses in each condition by age groups in experiment 1.

	Youngest third	Middle third	Oldest third
Blicket Condition	38.46	41.03	55.29
	(49.29)	(49.83)	(50.39)
	*n* = 39	*n* = 39	*n* = 38
	*M*_age_ = 58.84 months	*M*_age_ = 84.65 months	*M*_age_ = 108.17 months
Butterfly condition	26.19	38.10	59.52
	(44.50)	(49.15)	(49.68)
	*n* = 42	*n* = 42	*n* = 42
	*M*_age_ = 58.76 months	*M*_age_ = 80.44 months	*M*_age_ = 108.44 months

We broke the two conditions into three age groups shown in Table 1. These groups roughly corresponded to 4- and 5-year-olds, 6- and 7-year-olds, and 8–10-year-olds. In both conditions, the two younger groups responded no different from chance (33%, because we presented 3 answer choices), all Binomial tests, *p* > 0.19. The older group responded differently from chance, Binomial tests, *p* = 0.007 in the blicket condition and *p* < 0.001 in the butterfly condition.

### Justifications

As noted above, only 39 of the children in the blicket condition were asked to justify their responses (compared to 125 of the children in the butterfly condition), because this question was added partway through data collection. Fifteen children in the blicket condition (38.5%) and 38 children in the butterfly condition (30.4%) provided relevant justifications. This was not a significantly different ratio, χ^2^(1, *N* = 164) = 0.35, *p* = 0.35. Additionally, 9 children in the blicket condition (23.1%) and 25 in the butterfly condition (20.0%) said “I don’t know,” said that they were guessing, or made no response to this question; this response tendency also did not significantly differ by condition χ^2^(1, *N* = 164) = 0.17, *p* = 0.68.

To examine any potential links between children’s justifications and their performance, we conducted a binary logistic regression on whether children generated a relevant justification based on their age, condition, and whether they responded correctly on the test question. The overall model was significant, χ^2^(3) = 27.08, *p* < 0.001. Age predicted a unique proportion of variance, β = 0.04, SE = 0.01, Wald χ^2^ = 16.85, *p* < 0.001. Condition and performance on the test question were not significant in this model.

### Open-Ended Experimentation

After children justified their choice, they were allowed to try any combination of blocks/flower on the machine/in the field. Because different children tried different numbers of combinations, we only examined children’s first attempts. Our primary goal with this question was to explore whether children chose the correct combination of orange and blue, regardless of whether they had chosen this pair at test. Although this task was not designed to directly probe children’s predictive abilities, we speculated that this response tendency could indicate some implicit understanding of how the machine worked, even in the absence of a correct explicit response (see e.g., Cook et al., 2011).

Ninety-five children in the blicket condition and 119 children in the butterfly condition tried at least one combination of stimuli. Of these children, 38% in the blicket condition while only 24% in the butterfly condition tried the combination orange and blue (i.e., the correct response to the test question), a significant difference, χ^2^(1, *N* = 214) = 5.20, *p* = 0.02, Phi = −0.16.

We performed a binary logistic regression examining whether children tried the correct pair of stimuli, looking at age, condition, and whether children chose the correct response on the test trial. The overall model was significant, χ^2^(3) = 12.72, *p* = 0.005. Condition explained unique variance, with children in the Blicket condition being more likely than children in the Butterfly condition to try the orange/blue combination, β = −0.66, SE = 0.31, Wald χ^2^ = 4.63, *p* = 0.03. Correct responding on the test trial also explained unique variance; children who responded correctly on the test trial were significantly more likely to choose the orange/blue pair to test in their open-ended exploration, β = 0.86, SE = 0.32, Wald χ^2^ = 7.38, *p* = 0.007. Age did not explain unique variance, β = 0.006, SE = 0.007, Wald χ^2^ = 0.56, *p* = 0.45. We then ran a separate model with only condition, correct responding and the interaction between them. This model was a better fit (as indicated by a lower BIC value, 261.38 vs. 264.88), and indicated that the interaction between condition and correct responding was also significant, β = 1.26, SE = 0.63, Wald χ^2^ = 3.99, *p* = 0.046. Specifically, in the Blicket condition, children were more likely to try the orange/blue pair if they had chosen it at test (56% vs. 23% of the time). This difference was not as great in the Butterfly condition (25% vs. 22%).

### Discussion

Four- to 10-year-olds were given one of two kinds of diagnostic reasoning tasks. The first was a replication of the procedure used by Sobel et al. (2017), in which children had to make inferences about an interactive causal structure. The second was an inferential problem with the same causal structure, but different content – about biological instead of physical mechanisms. We generally replicated the earlier results using the blicket detector, and showed that children generated responses to the butterfly version of the same problem with a similar developmental trajectory. In many ways, this investigation parallels findings by Schulz and Gopnik (2004), who showed that preschoolers use the same domain-general formal principles of causal inference when reasoning about physical and biological content.

Unlike children’s performance with the main test question, the combinations of blocks that children chose to try in response to our open-ended prompt differed between the two conditions: Children in the Blicket condition were more likely to choose the correct (orange/blue) combination. Children in this condition who chose to try the correct combination were also more likely to have chosen this combination at test than in the Butterfly condition, potentially implying that they were verifying their choice. Because these choices reflect children’s predictive (rather than diagnostic) abilities, which were not the focus of this investigation, we hesitate to draw any strong conclusions about this result.

Finally, due to a change in procedure, only a subset of children in the Blicket condition in Experiment 1 was asked to justify their responses. We thought it critical to replicate this procedure to ensure the robustness of our findings about children’s justifications. Experiment 2 does so, while also presenting the two conditions as a within-subject manipulation. This allowed us to try to replicate the findings of Experiment 1 while controlling for individual-level variance and examining whether children notice the similarities between the two tasks.

## Experiment 2

### Method

#### Participants

The final sample included 103 children between the ages of 4–10 (55 girls, 48 boys, M_age_ = 82.93 months, SD_age_ = 22.33 months) from preschools, elementary schools, and after-school programs. As there is not an agreed-upon protocol for *a priori* power analysis for GEE, we aimed to test as many children as we could at our participating schools and to roughly match the sample size of Experiment 1. Twelve additional participants were tested, but not included: One did not respond to any questions, and for the other eleven, there were errors with the activation of the machine. In terms of race, 53 families identified as White, 34 identified as African American, 7 identified as multiracial, 1 identified as American Indian or Alaskan Native, 2 identified as Asian, and 6 did not provide this information. In terms of ethnicity, 9 families identified as Hispanic or Latino, 31 identified as not Hispanic or Latino and 63 did not provide this information.

#### Materials and Procedure

The same materials used in Experiment 1 were used in Experiment 2. The only difference in procedure was that Experiment 2 ran the two tasks one after the other (order counterbalanced). Additionally, in Experiment 2, we asked all participants to justify their answer choices. Finally, following children’s completion of their second task, we added a question to probe for whether they noticed anything similar about the two tasks: “We just played the machine [butterfly] game. Do you remember before when we played the butterfly [machine] game? Was anything the same about the machine game and the butterfly game?” We did this to investigate whether any children would code the similarity between the tasks as being deeper than just surface-level, and if their noticing of structural similarities between the tasks might relate to better performance.

#### Coding

We used the same coding for the main test questions, children’s justifications, and children’s first open-choice combination as in Experiment 1. Coding for the justifications was again done by two research assistants, blind to children’s age and gender, but not blind to whether they were coding a blicket or butterfly response (coding for the blicket trials was done at a separate time than coding for the butterfly trials). Agreement for the butterfly trials was 91% (Kappa = 0.81). Agreement for the blicket trials was 95% (Kappa = 0.89). Disagreements were resolved through discussion with the second author.

For the similarity question, we scored children’s responses as 1 if they said they noticed that something was the same about the two tasks and 0 otherwise. For children who recognized the similarity, we coded their reasons as either *causal* or *perceptual*. Causal responses mentioned the similarity of causal structure of the two games, for example, “both of them turned green when you put all of them.” Perceptual responses mentioned the similarity of any perceptual aspect of the two games, for example, “both were red and green.” Two coders independently coded 10% of the sample and agreement was 100%. A single coder then coded the remainder of the sample.

### Results

Preliminary analyses revealed no effects of order (i.e., receiving the blicket or butterfly version first) on responses to either test question, both χ^2^(1, *N* = 103)-values < 0.47, both *p*-values > 0.61. Similarly, there was no difference between girls and boys in their response to either test question, χ^2^(1, *N* = 103)-values < 0.70, both *p*-values > 0.41. As a result, these variables were not considered further. We constructed a General Estimating Equation (GEE) with an independent working correlation matrix and a binomial distribution (following Zeger and Liang, 1986; Zeger et al., 1988). This accounted for the within-subject nature of our procedure. Correct performance was the dependent variable, and age and condition were the independent variables. This model revealed a significant main effect of age, Wald χ^2^ = 19.86, *p* < 0.001, but not a significant effect of condition, Wald χ^2^ = 0.33, *p* = 0.56.

Overall, the sample’s performance on the blicket measure (46% accurate) was better than what would be expected by chance (33%), Binomial test, *p* = *0.005*, as was performance on the butterfly measure (42%), Binomial test, *p* = *0.04*. Given the results of Experiment 1, we separated our sample into three roughly equal groups based on age. These results are shown in Table 2. The youngest group (roughly 4–5-year-olds, *M*_age_ = 57.03 months, Range 46.20–68.63 months) responded no different from chance on either measure, Binomial tests, both *p*-values > 0.15. The middle group (roughly 6–7-year-olds, *M*_age_ = 83.89 months, Range 69.57–96.20 months) also responded no different from chance on either measure, Binomial tests, both *p*-values > 0.15. The oldest group (roughly 8–10-year-olds, *M*_age_ = 107.84 months, Range 96.93 132.87 months) responded above chance on both measures, Binomial tests, *p* = 0.002 for the butterfly measure and *p* < 0.001 for the blicket measure.

**TABLE 2:** Proportion of correct responses in each condition by age groups in experiment 2.

	Youngest third (*n* = 34)	Middle third (*n* = 35)	Oldest third (*n* = 34)
Blicket condition	23.53 (43.06)	42.86 (50.21)	70.59 (46.25)
Butterfly condition	29.41 (46.25)	37.14 (49.02)	58.82 (49.96)

#### Justifications

Children generated relevant justifications on 37% of the butterfly trials and 31% of the blicket trials. A new General Estimating Equation was built to analyze appropriate responses on each trial, with age, condition, and whether children responded correctly on the test question as dependent variables. This model revealed a main effect of age, Wald χ^2^ = 5.25, *p* = 0.02. Condition was not significant, Wald χ^2^ = 1.55, *p* = 0.21. The effect of correct response on the test trials did not reach significance, but children who had responded correctly were marginally more likely to generate relevant justifications, Wald χ^2^ = 3.00, *p* = 0.08.

#### Open-Ended Experimentation

We again examined how many children selected the orange/blue (correct) combination as the first combination they wanted to try. To parallel the analysis from Experiment 1, we ran a GEE on whether children tried the correct combination in each trial, with age, condition, and correct responding as dependent variables. This resulted in a main effect of condition, β = −0.98, SE = 0.35, Wald χ^2^ = 8.01, *p* = 0.005 and a main effect of correct responding, β = 1.20, SE = 0.39, Wald χ^2^ = 9.32, *p* = 0.002, but not a main effect of age^2^. Of importance is that the main effect of condition was the opposite of Experiment 1. Here, for the blicket condition, 13% of children’s attempts were orange/blue, compared to 25% for the butterfly condition. This was a significant difference in ratios, McNemar χ^2^(1, *N* = 103) = 6.50, *p* = 0.01.

In terms of the effect of correct responding, in the butterfly condition, children who responded correctly on the test question were more likely to choose to try the correct combination in the butterfly condition (33%) than children who did not respond correctly (20%). Similarly, in the blicket condition, children who responded correctly on the test question were more likely to choose to try the correct combination (29%) than children who did not (0%). Only in the blicket condition did this difference reach statistical significance, Fisher Exact Test, *p* = 0.18 and *p* < 0.001.

#### Similarity Question

Three children did not respond to the question about similarity. Of those who did, 82% of the participants said that they noticed some similarity between the two tasks, significantly more than chance, Binomial Test, *p* < 0.001. This response did not correlate with children’s age, *r*_s_(98) = 0.10, *p* = 0.32. When asked to justify why children believed the two trials were similar, 70% mentioned perceptual similarity whereas 30% generated a causal justification. Generating a justification of a particular type did not significantly correlate with age, nor did it correlate with performance on either the blicket or butterfly trial, all *r*_s_(80)-values < | 0.14|, all *p-*values > 0.23.

#### Comparisons to Experiment 1

Finally, we tested whether children responded differently on either the butterfly or the blicket conditions between Experiment 1 and 2. We found no difference in performance on either the blicket conditions across the studies (46% in each study), χ^2^(1) < 0.001, *p* = 0.99, or on the butterfly conditions (42% in each study), χ^2^(1) = 0.001, *p* = 0.98. This suggests that the within-subject nature of this experiment did not affect performance compared to a between-subject version of the same measure.

### Discussion

Experiment 2 found similar results to Experiment 1, whereby performance on both tasks improved with age, though only children in the oldest group performed at above-chance levels. We also found no differences between performance on these two tasks for any age group, either in terms of their responses to the test questions or in terms of their justifications for these responses. As in Experiment 1, 4- to 7-year-olds mostly responded at chance levels of performance, while 8- to 10-year-olds were generally above chance at making the appropriate diagnostic inference.

When examining which combinations of blocks children chose to try in the two causal systems, we did find a difference between the conditions, but in the opposite direction from Experiment 1. Here, children were more likely to try the correct answer in response to our open-ended prompt (orange/blue) for the Butterfly task than for the Blicket task. Additionally, for both tasks (though only statistically significantly for the Blicket task), children were more likely to try the orange and blue blocks if they had previously chosen this pair at test. Future work should look more directly at this relation between children’s diagnostic and predictive reasoning, as the current studies were not designed to specifically capture this aspect of children’s thinking, and past work suggests that these two reasoning process are not symmetrical (see e.g., Kuhn et al., 2009; Fernbach et al., 2010).

Finally, for the question about similarity between the two procedures, most of the children (correctly) said that the two tasks were similar to each other, although only a minority of children articulated that this similarity was due to the underlying causal structure rather than to the perceptual features of the two tasks. Saying that the two tasks were similar to each other did not affect performance on either measure, nor did children’s reported reason for why the tasks were similar (for example, talking about the causal structure or the more superficial perceptual features).

The two experiments so far suggest that children show similar diagnostic reasoning abilities across different domains of content. However, the blicket and butterfly measures were equivalent in a variety of ways, beyond just the causal structure. Both tasks involved diagnosing a set of causal relations, and among those causal relations, children had to track different levels of a single feature (color) to discern among potential causes. While the causal system was novel, in neither condition did children hear any information that was potentially unfamiliar or that required additional definitions or background knowledge – that is, the potential causes were all easily identifiable and comprehensible. All of these factors potentially facilitated children’s diagnostic inference.

## Experiment 3

In Experiment 3, we introduced a novel measure that used the same interactive structure used in Experiments 1 and 2, but that couched the problem in a more realistic scientific framework, increasing the information processing demands of the task. Instead of asking children to use colors make inferences about a set of causal relations, we asked children to use a set of biological features to infer category membership (whether an exemplar was a particular kind of dinosaur). The four features were presented as an interactive structure: Having both feature A and B meant that the example was one kind of dinosaur; having only A or only B meant that the example was a second kind; and having neither meant that the example was a third. However, each feature was unique (unlike Experiments 1 and 2, which used different levels of the color feature) and not directly observable (as was the case with the individual blocks or flowers). Thus, although Experiment 3 in some ways presents the same structure as Experiments 1 and 2, it differs in many important ways, which serve to make it a better test of the kinds of diagnostic inferences that children are asked to make in classrooms and in real life.

The fundamental question behind our investigation so far has been to examine whether children engage in diagnostic inference differently across domains. The first two experiments support the hypothesis that there is little difference between the inferences that children can make in the physical and biological domains. Biological relations, however, can be much more complex than what we manipulated in Experiments 1 and 2. Experiment 3 thus presents a more stringent test of our research question.

### Method

#### Participants

The final sample included 110 children between the ages of 4–10 (52 girls, 58 boys, M_age_ = 80.08 months, SD_age_ = 22.62 months), again aiming to roughly match the sample size of Experiment 1. These children were primarily recruited from and tested in local museums (*n* = 107), and a minority were tested at our lab or in a preschool (*n* = 3). We additionally tested 2 participants who were not included in the final sample. One child was a 3-year-old; the other refused to respond to questions.

The demographics of this sample were as follows: 68 families identified as White, 9 identified as African American, 3 identified as multiracial, 7 identified as Asian, and 23 did not provide this information. In addition, 12 families identified as Hispanic or Latino, 13 identified as not Hispanic or Latino and 85 did not provide this information.

#### Materials

**FIGURE 3:**
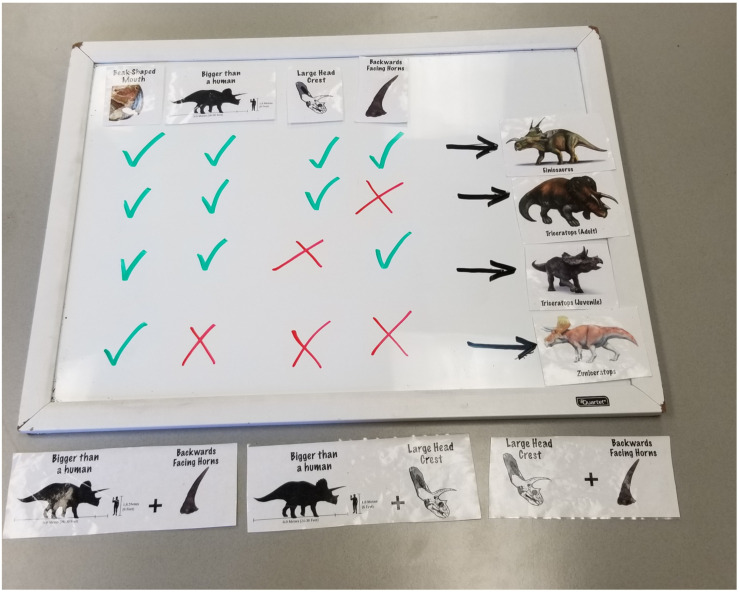
Completed grid for the dinosaur task. The experimenter would first explain and place the traits as column headers, then add checks and Xs with whiteboard markers to characterize the dinosaurs. The three answer choices appear at the bottom.

We used a whiteboard (22.75 inches wide x 17 inches high) to present a grid that showed which combinations of traits were characteristic of which kinds of dinosaurs (see Figure 3 and the Procedure section for more details). We created a set of 8 laminated pictures, one for each of four traits (e.g., having a beak-shaped mouth) and one for each of four dinosaurs (e.g., Einiosaurus). These could be stuck to the whiteboard using Velcro dots during the procedure to provide a visual aid for our explanations. We also used a green and a red whiteboard marker to indicate the presence (green check) or absence (red X) of each trait.

#### Procedure

To create the dinosaur version of the diagnostic reasoning task, we selected three dinosaurs from the family of ceratopsians (horned-headed dinosaurs): Einiosaurus, Triceratops, and Zuniceratops. We also selected four traits that these dinosaurs could have: mouth shape (beak-shaped or not), size (larger or smaller than a human when fully grown), head crest (large or small), and horn direction (backwards-facing or forwards-facing). As in the other two versions of this task, children saw four demonstrations of how different traits combined to characterize the three types of dinosaurs. These demonstrations were presented in one of two orders. Order 1 first presented Einiosaurus, then adult Triceratops, then baby Triceratops, then Zuniceratops; Order 2 presented the dinosaurs in the reverse order. We here use Order 1 to illustrate the procedure.

Children were told that they would play a dinosaur game to learn about the different traits that dinosaurs can have. They were then oriented to the grid on the whiteboard and to the four traits that served as column headers (see Figure 3). An experimenter first told children that some dinosaurs belong to the ceratopsian family, which means that they have beak-shaped mouths. The experimenter showed the child the picture card that illustrated a beak-shaped mouth, then stuck it to the first column on the whiteboard. The same was done for the next three traits: being larger than a human when fully grown, having a large head crest, and having backwards-facing horns. As the experimenter described each trait, she showed the child a picture of that trait and then stuck it to the whiteboard to fill out the column headers. These traits were always presented in the same order.

Then, children were told that they would see some dinosaurs, and that some of these dinosaurs have these traits while others do not. Order 1 first introduced Einiosaurus. The experimenter brought out the picture card for this dinosaur and explained that Einiosaurus has all four traits: It has a beak-shaped mouth, is larger than a human when fully grown, has a large head crest, and has backwards-facing horns. This dinosaur was thus the parallel of placing all four blocks on the machine or planting all four flowers in the field. As the experimenter named each of the four traits, she used the green whiteboard marker to put a green check under each column of the first row of the grid on the whiteboard. At the end of this introduction, the experimenter repeated the combination of traits: “So if a dinosaur has a beak-like mouth and is bigger than a human when it’s fully grown and has a big head crest and has backwards facing horns, that means it is an Einiosaurus.” She then stuck the Einiosaurus picture card to the right of the first row.

The same procedure was repeated for the other three dinosaurs, using green checks when a dinosaur had a trait and a red Xs when a dinosaur lacked a trait (see Figure 3). Adult Triceratops has a beak-shaped mouth, is larger than a human when fully grown, and has a large head crest, but it does not have backwards-facing horns; its horns face forward. Baby Triceratops has a beak-shaped mouth, is larger than a human when fully grown, and has backwards-facing horns, but it has a small head crest. Because Triceratops changes its head crest size and horn direction as it matures (Horner and Goodwin, 2006), it served as the equivalent of the red activation on the blicket machine or the red flowers in the butterfly field: Two different combinations of three traits led to the same type of dinosaur, just as two different combinations of three blocks/flowers led to a red light/red butterflies.

The fourth dinosaur, Zuniceratops, has only one of the four traits: a beak-shaped mouth. But it is smaller than a human when fully grown, lacks a head crest, and has forward-facing horns.

At the end of this demonstration phase, children could see the full set of trait combinations on the grid on the whiteboard (Figure 3). To frame the test question, children were told that paleontologists have found a new set of fossils that they are sure is an Einiosaurus. But the paleontologists used only two traits to figure that out. Children were asked to choose which pair of traits the paleontologists used to know for sure that the fossils they found belonged to an Einiosaurus: (a) being bigger than a human and having backwards-facing horns, (b) being bigger than a human and having a large head crest, or (c) having backwards-facing horns and having a large head crest. In parallel to the other two versions of this task, the correct answer is c, since only the Einiosaurus has both of these traits.

These answer choices were accompanied by illustrations that combined the relevant pictures from the column headers on the whiteboard (see bottom of Figure 3). The answer choices were presented in a random order for each child. The corresponding picture cards were placed on the table in front of the child as the experimenter described each one. As in the previous versions of this task, after choosing a response option, children were asked to justify their choice.

#### Coding

Answers of “backwards-facing horns and having a large head crest” were coded as correct; the other two answers were coded as incorrect. We coded children’s justifications as *relevant* when they mentioned the different traits that the dinosaurs could have or when they made comparisons between the dinosaurs (e.g., “well, if you think it’s this [pointing to Einiosaurus] bigger than a human, it’s pretty easy, and a large headcrest is only on two of them, which I’ve seen so that makes it, so that means narrowing the options and so from there we can just look at the details and probably try to figure it out.”). We coded children’s justifications as *irrelevant* when they did not provide any substantive information (e.g., “Because it is bigger than a human and because dinosaurs are so big”) or when they said “I don’t know.” Justifications were coded by two research assistants who were blind to children’s age and gender. These two coders considered 89% of the dataset. Agreement was 89% (Kappa = 0.77). Disagreements were resolved through discussion with the third author and then the first coder coded the rest of the data.

### Results

Preliminary analyses showed that the order in which information was presented did not affect responses, χ^2^(1, *N* = 110) = 0.04, *p* = 0.85. Preliminary analyses also showed that girls and boys did not differ in their responses, χ^2^(1, *N* = 110) = 0.01, *p* = 0.94. As a result, we do not consider these factors further.

Overall, children responded correctly 27% of the time, not significantly greater than chance, Binomial test, *p* = 0.20. We tested for age effects using a general linear model with a binomial logistic distribution, as in Experiment 1. The overall model was not significant, χ^2^(1) = 3.17, *p* = 0.08, nor was the individual effect of age, B = −0.02, SE = 0.01, 95% CI [−0.04, 0.00], Wald χ^2^(1) = 3.11, *p* = 0.08. As a way of initially contrasting performance in this condition to that of the other conditions, we broke the sample into thirds based on age, as we did in the previous two studies. No age group’s performance was above chance responding (youngest: 25%; middle: 21%; oldest: 36%; all Binomial tests, *p*-values > 0.14).

We examined children’s justifications by conducting a general linear model with a binary logistic distribution, predicting whether children generated a relevant justification based on their age and whether they responded correctly on the test question. The overall model was significant, χ^2^(2) = 10.35, *p* = 0.006. Age was a significant predictor, Wald χ^2^(1) = 9.14, *p* = 0.003. Correct judgments on the test question was not a significant predictor, Wald χ^2^(1) < 0.01, *p* = 0.99.

To contrast performance with the blicket and butterfly conditions in Experiment 1, we again used a general linear model with a binomial logistic distribution, examining the role of age (in months) and task version (blicket, butterfly, dinosaur). The overall model was significant, χ^2^(3) = 22.54, *p* < 0.001. This analysis revealed a main effect of condition, Wald χ^2^(2) = 7.12, *p* = 0.03 and a main effect of age, Wald χ^2^(1) = 13.60, *p* < 0.001. Overall, performance on the dinosaur condition (27% correct) was worse than performance on the blicket condition (45% correct), B = −0.74, SE = 0.29, 95% CI [−1.30, −0.17], Wald χ^2^(1) = 6.46, *p* = 0.01, and performance on the butterfly condition (41%), B = −0.61, SE = 0.29, 95% CI [−1.17, −0.05], Wald χ^2^(1) = 4.52, *p* = 0.03.

### Discussion

On this dinosaur task, we found no improvement in performance with age, and even the oldest age group tested responded at chance levels. Although the underlying reasoning structure of the dinosaur task was identical to that of the blicket and butterfly tasks presented in Experiments 1 and 2, children had more difficulty solving this version of the problem. As prior work has shown that children understand the role of biological features in categorization (Keil, 1989; Carey, 1995; Slaughter and Lyons, 2003), we believe that this difference in performance is due to other features of our task, most importantly the interaction between the task’s surface features and its underlying structure: The use of dinosaurs made it appear as though domain-specific knowledge was necessary to solve the task, although this was not the case. We discuss this issue further in the General Discussion, recalling that our goal with this experiment was to begin to explore how more realistic science content can affect children’s reasoning, and not to investigate the impact of individual task features on children’s performance.

## General Discussion

The current study examined whether children possessed similar diagnostic reasoning abilities across different domains of knowledge. In Experiment 1, we replicated previous findings that suggested children could engage in diagnostic reasoning about an interactive causal structure by age 7 (Sobel et al., 2017). We also extended this finding to another domain of knowledge: biology. We demonstrated that children’s diagnostic reasoning is not limited to the blicket detector paradigm; children performed similarly on both versions of the task. Using a within-subject design, instead of a between-subject design, Experiment 2 replicated the findings of Experiment 1, as well as corrected for an error in data collection regarding children’s justifications of their inferences. These results imply that the mere presence of domain-specific features does not impact children’s abilities to reason diagnostically. Further, these results confirm that this kind of diagnostic reasoning emerges around age 7, possibly because younger children lack the capacity to coordinate the high level of uncertainty about the efficacy of individual variables presented by our task (e.g., Erb and Sobel, 2014) or lack the metacognitive capacities to reflect on this uncertainty (Kuhn and Dean, 2004).

In contrast to the first two experiments, children performed much more poorly when asked to make diagnostic inferences about category membership of dinosaurs based on their features (Experiment 3). Here, although the underlying structure of the problem was the same as in Experiments 1 and 2, children did not show improvement with age, and even the oldest children we tested did not respond correctly above chance levels.

There were, of course, many important differences between the dinosaur version of the task presented in Experiment 3 and the tasks presented in Experiments 1 and 2, any of which (or their combination) could explain this difference in performance. Children in Experiment 3 had to track different features (e.g., size and shape) as relevant for category membership, as opposed to different levels of a single feature (color). In addition, there was a difference in degree of complexity of the outcomes: The dinosaur condition featured children learning the names of different (potentially novel) dinosaurs and their features, whereas the other conditions did not require the child to learn novel vocabulary or other scientific content. Children may also have differed in their level of domain-specific knowledge about biology in general or dinosaurs in particular, which may have affected the degree to which they believed that the task required such knowledge to solve. Future work should attempt to examine which of these features have the most impact on children’s reasoning abilities. But, crucially, it might not be possible for such investigations to disentangle whether children experience more difficulty in more realistic problems because of information processing demands or because of the realism itself; increasing the amount of domain-specific knowledge in a domain of reasoning will necessarily involve adding information to a given causal model.

Equally crucially, we would argue that the fact that the dinosaur version of the task was not precisely matched to the blicket and butterfly versions of the task is somewhat beside the point. Our goal in Experiment 3 was to instantiate a causal structure for which the developmental trajectory was well-understood (from work with blicket detectors) in a context that superficially resembled reasoning tasks that children see in classrooms or in prior work on diagnostic inferences (Kuhn and Dean, 2005; Kuhn et al., 2009). In doing so, although we modified more than just one aspect of the task, we were able to take an initial step toward reconciling conflicting conclusions from cognitive developmental psychology, claiming that even babies can reason diagnostically (e.g., Gopnik et al., 2004; Sobel and Kirkham, 2006), with work from education science, claiming that children struggle with diagnostic thinking until late elementary school and beyond (e.g., Klahr et al., 1993; Chen and Klahr, 1999).

In particular, the differences demonstrated here between the blicket and butterfly versions of the reasoning task and the dinosaur version have important implications for how the development of diagnostic reasoning is understood. Children’s performance on the blicket and butterfly versions of the task demonstrated that at least the older children in our sample (8- to 10-year-olds) have the reasoning skills necessary to diagnose the interactive causal system that we presented [as also shown by Koerber et al. (2015) and Sobel et al. (2017)]. On this basis, some have suggested that these children (and even younger children) are mature scientific thinkers, since diagnostic reasoning is an important component of scientific thinking (e.g., Cook et al., 2011; Gopnik and Wellman, 2012).

But there are many important methodological differences between the tasks used in those studies (and in our Experiments 1 and 2) and other investigations of scientific reasoning (and in our Experiment 3), such as understanding experimental design, engaging in hypothesis formation, and reflecting on the quality of data and what inferences those data support. Many of these differences have been discussed and investigated elsewhere (e.g., Sodian et al., 1991; Ruffman et al., 1993; Kuhn and Pearsall, 2000; Kuhn, 2007; Sobel et al., 2017) and are beyond the scope of the present investigation. Indeed, these additional reasoning capacities are likely only available to older children, and the continuing developmental trajectory of children’s scientific reasoning abilities may involve increasing coordination among these capacities and the kind of diagnostic reasoning abilities investigated in the current work. Rather, we wish to emphasize that prior work on scientific reasoning has tended to focus on making diagnostic inferences about domain-specific contexts, like those found in science classrooms (see e.g., NGSS Lead States, 2013). Because our Experiment 3 resembles such systems more closely, our results suggest that the information processing demands of such contexts (which come from many sources) may prevent children from demonstrating their existing capacities to reason about such systems. Children may have broad diagnostic reasoning capacities in the early elementary school years, but applying those capacities to the science classroom seems to only come later in development.

## Data Availability Statement

The datasets generated for this study are available on request to the corresponding author.

## Ethics Statement

The studies involving human participants were reviewed and approved by the Villanova University Institutional Review Board. Written informed consent to participate in this study was provided by the participants’ legal guardian/next of kin.

## Author Contributions

DW and DS conceptualized the study and secured funding. EC executed the design, collected the data for Studies 1 and 2, and wrote the first draft of the manuscript. DW executed the design and collected the data for Study 3. DS performed the statistical analyses. All authors then contributed to the editing process.

## Conflict of Interest

The authors declare that the research was conducted in the absence of any commercial or financial relationships that could be construed as a potential conflict of interest.

## Footnotes

1 We use this strategy throughout the analysis of all three experiments. We considered models with main effects only and models that included both main effects and interactions. We only report the latter if it was a better fitting model. We indicate where this is the case; in all other cases throughout these experiments, the main effects only model was the better-fitting model.

2 Because children who responded incorrectly on the test question never tried the correct combination on the machine, it was not possible to test the interaction, which was significant in Study 1.
